# Supplementary material for: The interactome of intact mitochondria by cross-linking mass spectrometry provides evidence for coexisting respiratory supercomplexes
Source: Mol Cell Proteomics. 2017 Dec 8;17(2):216–32. doi: 10.1074/mcp.RA117.000470 (PMC5795388; doi:10.1074/mcp.RA117.000470)
Supplement: Supplemental Data [file supp_RA117.000470_133922_0_supp_23978_fzffwf.zip › spectra_annotation/mito_spectra_annotation/434-1-6-1-6-1.pdf]

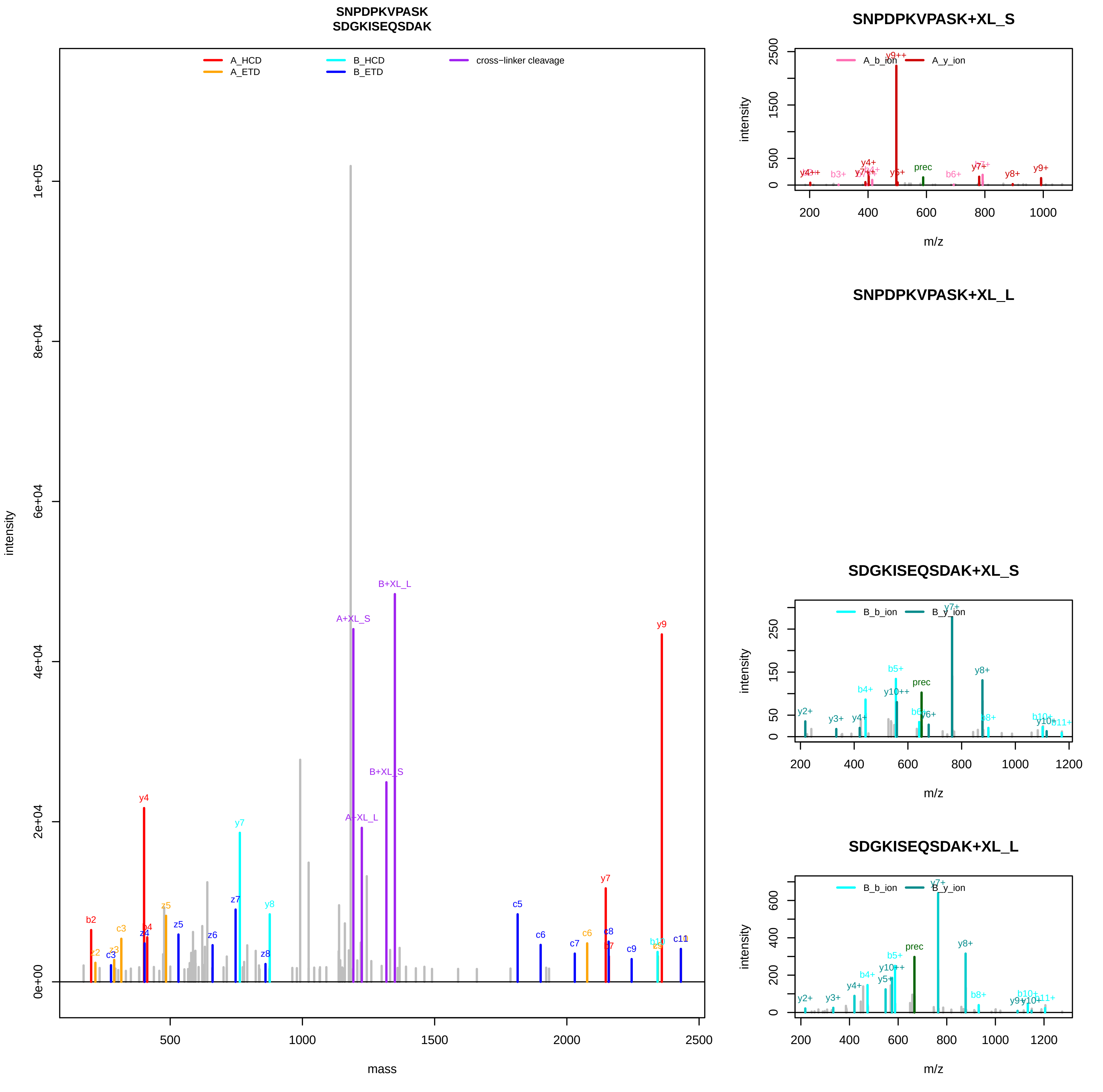
SNPDPKVPASK
SDGKISEQSDAK
A_HCD
A_ETD
B_HCD
B_ETD
cross−linker cleavage
intensity
mass
0e+00
2e+04
4e+04
6e+04
8e+04
1e+05
500
1000
1500
2000
2500
b2
z2
c3
z3
c3
y4
b4
z4
z5
z5
z6
z7
y7
y8
z8
A+XL_S
A+XL_L
B+XL_S
B+XL_L
y9
y7
b7
c5
c6
c6
c7
c8
c9
b10
c9
c11
SNPDPKVPASK+XL_S
A_b_ion
A_y_ion
y9++
y4+
y3++
b3+
y7++
b4+
y5+
prec
b6+
y7+
b7+
y8+
y9+
m/z
SNPDPKVPASK+XL_L
SDGKISEQSDAK+XL_S
B_b_ion
B_y_ion
y7+
y2+
y3+
y4+
b4+
b5+
y10++
prec
b6+
y6+
y8+
b8+
b10+
y10+
b11+
SDGKISEQSDAK+XL_L
y4+
b4+
b5+
y10++
y5+
y9+
y10+
200
400
600
800
1000
1200
0
50
150
250
500
1500
2500
